# Renal Cell Carcinoma Associated with Xp11.2 Translocation/TFE3 Gene Fusions: Clinical Features, Treatments and Prognosis

**DOI:** 10.1371/journal.pone.0166897

**Published:** 2016-11-28

**Authors:** Ning Liu, Zhen Wang, Weidong Gan, Lei Xiong, Baolei Miao, Xiancheng Chen, Hongqian Guo, Dongmei Li

**Affiliations:** 1 Department of Urology, Nanjing Drum Tower Hospital, The Affiliated Hospital of Nanjing University Medical School, Nanjing, Jiangsu, China; 2 Immunology and Reproduction Biology Laboratory & State Key Laboratory of Analytical Chemistry for Life Science, Medical School, Nanjing University, Nanjing, Jiangsu, China; 3 Jiangsu Key Laboratory of Molecular Medicine, Nanjing University, Nanjing, Jiangsu, China; National Institute of Health, UNITED STATES

## Abstract

To investigate the clinical characteristics, treatments and prognosis of renal cell carcinoma associated with Xp11.2 translocation/TFE3 gene fusions (Xp11.2 tRCC), the epidemiological features and treatment results of 34 cases of Xp11.2 tRCC, which were diagnosed by immunohistochemistry staining of TFE3 and fluorescence in situ hybridization at our center, were retrospectively reviewed. The 34 patients included 21 females and 13 males aged 3 to 64 years (median age: 27 years). Four patients were children or adolescents (<18 years of age), and 26 patients were young or middle-aged adults (18–45 years). Radical nephrectomy was performed on 25 patients. Laparoscopic nephron-sparing surgery was performed on 9 patients who presented with an isolated mass with a small diameter (<7 cm) and well-defined boundary on computed tomography imaging. Postoperative staging showed that 25 cases (73.53%) were at stage I/II, while 9 cases (26.47%) were at stage III/IV. All stage I/II patients received a favorable prognosis with a three-year overall survival rate of 100%, including the patients who underwent laparoscopic nephron-sparing surgery. With the exception of 2 children, the other 7 stage III/IV patients died or developed recurrence with a median follow-up of 29 months. On univariate analysis, maximum diameter, adjuvant treatment, TNM stage, lymph node metastasis, inferior vena cava tumor thrombosis and tumor boundary were identified as statistically significant factors impacting survival (P<0.05). Multivariate analysis indicated that TNM stage and inferior vena cava tumor thrombosis were independent prognostic factors (P<0.05). In conclusion, Xp11.2 tRCC is a rare subtype of renal cell carcinoma that mainly occurs in young females. Nephron-sparing surgery was confirmed effective preliminarily in the treatment of small Xp11.2 tRCCs with clear rims. Advanced TNM stage and inferior vena cava tumor thrombosis were associated with poor prognosis.

## Introduction

Since being recognized as a distinct entity by the World Health Organization (WHO) in 2004 [1], Xp11.2 translocation renal cell carcinoma (Xp11.2 tRCC) has attracted broad attention [2–5]. The term of Xp11.2 tRCC derived from several different chromosomal translocations of Xp11.2 breakpoints and formatting of TFE3 fusion gene, which resulted in a significant overexpression of TFE3 protein in tumor cells. In recent years, renal cell carcinoma associated with t(6;11)(p21;q12)/TFEB gene fusions has been found to share similar pathology, epidemiology and genetics characteristics with Xp11.2 tRCC[6]. At the same time, both TFE3 and TFEB are members of microphthalmia-associated transcription (MiT) factor family. On the basis of these findings, WHO in 2016 newly designated Xp11.2 tRCC as MiT family translocation renal cell carcinoma [7].

Microscopically, Xp11.2 tRCC is similar to clear cell renal cell carcinoma (CCRCC) or papillary renal cell carcinoma (PRCC) [4, 8], which makes it difficult for pathologists to distinguish Xp11.2 tRCC from other tumor types by histological characteristics. Although immunohistochemical staining for TFE3 (TFE3-IHC) serves as the basic method for the diagnosis of Xp11.2 tRCC, numerous reports have shown that TFE3-IHC has fairly high false-positive rates and low predictive values, which results in misdiagnoses in patients [4, 9–11]. To date, TFE3 break-apart fluorescence in situ hybridization (FISH) is regarded as the best method to diagnose Xp11.2 tRCC due to its advantages of high sensitivity and specificity [2, 12–15]. In the current study, 34 cases of Xp11.2 tRCC were diagnosed using a TFE3 break-apart FISH probe. To assess whether ASPL-TFE3 RCC, one of the subtypes of Xp11.2 tRCC with a fusion pattern of t(x;17)(p11.2;q25), showed more aggressive progress than other subtypes, we used an ASPL-TFE3 dual-fusion FISH probe for the diagnosis of ASPL-TFE3 RCC. Both the TFE3 break-apart FISH probe and ASPL-TFE3 dual-fusion FISH probe were demonstrated to identify the TFE3 and ASPL-TFE3 fusion genes, respectively, in our previous investigations [11, 15].

Despite its low incidence, Xp11.2 RCC is more harmful than conventional RCC because the majority of patients present at advanced stages and invasive clinical courses [2–5, 16]. Surgical treatment, especially radical nephrectomy (RN), remains the most common strategy to treat Xp11.2 tRCC. The implementation of nephron-sparing surgery (NSS) in Xp11.2 RCC is rarely reported, although it has been confirmed safe and effective in conventional RCC by several large retrospective studies [17, 18]. Herein, the outcomes of patients receiving RN or NSS were compared to ascertain the effectiveness of NSS for these potentially aggressive tumors.

## Patients and Methods

### Patients and diagnosed methods

The RCC patients were reviewed at Nanjing Drum Tower Hospital from January 2007 to February 2016, and the study was conducted from February to June 2016. All procedures were approved by the Medical Ethics Committee for human Experiments of Nanjing Drum Tower Hospital. This study involved the preoperative computed tomography (CT) characteristics, medical records, follow-ups and outcomes of patients. IHC staining was performed on formalin-fixed paraffin-embedded tissue sections with a TFE3 antibody, and 2 (+) to 3 (+) nuclear TFE3 immunoreactivity in more than 10% of cells was considered positive. Polyclonal break-apart probes for TFE3 gene rearrangement at the Xp11.2 region were utilized on samples from patients who had a positive TFE3-IHC result on formalin-fixed paraffin-embedded tissue microarray slides. Of the 1,239 RCC patients, 82 cases showed positive reaction to TFE3-IHC, and 34 cases were eventually diagnosed as Xp11.2 tRCC by FISH. With the exception of one young female patient who lost to follow-up at the 88^th^ postoperative month, the other 33 cases were followed over a period ranging from 3 to 104 months. The clinicopathological characteristics and follow-up information of these patients are shown in Table 1. To ascertain specific types of genetic changes in tumor cells, 23 cases (case 1 to case 23 in Table 1) were subjected to the ASPL-TFE3 dual-fusion FISH assay, but case 24 to case 34 were not achieved due to the absent of tissue sections. All the patients have signed in informed written consents to have their medical record data used in research.

**Table 1:** Clinical data of 34 cases of Xp 11.2 tRCC.

Case	Age (years)/Sex/Laterality	Symptom	Tumor size (cm)	Operation	Final diagnosis	ACJJ stage	Adjuvant therapy	Follow-up (months) and outcome
1	21/F/R	Symptomless	4	LRN	ASPL-TFE3 RCC	pT1aN0M0, I	None	60, Normal
2	25/M/R	Gross hematuria, Flank pain	7.1	LRN	ASPL-TFE3 RCC	pT2aN0M0, II	IT	18, Normal
3	35/M/R	Symptomless	6	LRN	Xp11.2 RCC	pT1bN0M0, I	TMT	50, Lung metastasis in 11 months, stable now
4	26/M/L	Symptomless	3.7	ORN	Xp11.2 RCC	pT1aN0M0, I	TMT	74, Normal
5	39/F/R	Symptomless	13	ORN+VCTER	Xp11.2 RCC	pT3bN1M0, III	TMT	25, Died of liver and brain metastasis
6	46/F/R	Flank pain, Abdominal mass	5.8	ORN+VCTER	Xp11.2 RCC	pT3cN0M0, III	TMT	15, Died of lung metastasis
7	22/F/R	Gross hematuria	3.9	LRN	ASPL-TFE3 RCC	pT1aN1M0, III	TMT	62, Died of bone metastasis
8	26/F/R	Gross hematuria	5	LRN	Xp11.2 RCC	pT1bN0M0, I	None	96, Normal
9	7/M/L	Gross hematuria	3	ORN	ASPL-TFE3 RCC	pT1aN0M0, I	None	104, Normal
10	36/F/R	Gross hematuria	8.6	ORN+VCTER	ASPL-TFE3 RCC	pT3cN1M0, III	TMT	33, Died of liver metastasis
11	30/F/R	Symptomless	3.2	RA+LNSS	Xp11.2 RCC	pT1aNxM0, I	TMT	63, Normal
12	7/M/L	Gross hematuria, Abdominal mass	10	ORN	Xp11.2 RCC	pT4N1M0, IV	None	65, Normal
13	25/F/L	Symptomless	3.8	LRN	Xp11.2 RCC	pT1aN0M0, I	IT	58, Normal
14	24/F/R	Symptomless	3.9	LRN	Xp11.2 RCC	pT1aN0M0, I	IT	42, Normal
15	51/F/R	Symptomless	5	LNSS	Xp11.2 RCC	pT1bNxM0, I	IT	53, Normal
16	27/F/R	Gross hematuria, Flank pain	6	LRN	Xp11.2 RCC	pT1bN0M0, I	IT	53, Normal
17	26/M/L	Symptomless	3.7	LNSS	Xp11.2 RCC	pT1aN0M0, I	IT	18, Normal
18	3/F/R	Gross hematuria	4	ORN	Xp11.2 RCC	pT1aN1M0, III	None	71, Normal
19	11/F/R	Gross hematuria, Abdominal mass	5.6	ORN	Xp11.2 RCC	pT1bN0M0, I	None	88, Lost
20	40/M/L	Symptomless	3.9	LRN	Xp11.2 RCC	pT1aN0M0, I	None	37, Normal
21	19/F/L	Symptomless	5	LRN	Xp11.2 RCC	pT1bN0M0, I	None	24, Normal
22	38/M/R	Gross hematuria, Flank pain	3	LRN	ASPL-TFE3 RCC	pT1aN0M0, I	IT	28, Normal
23	29/M/L	Symptomless	3.5	LNSS	Xp11.2 RCC	pT1aN0M0, I	IT	10, Normal
24	25/F/R	Gross hematuria	8.1	LRN	Xp11.2 RCC	pT2aN0M0, II	None	91, Normal
25	27/M/L	Symptomless	8.5	LRN	Xp11.2 RCC	pT3aN0M0, III	TMT	24, Recurred in 16 months
26	22/F/R	Gross hematuria	5	LRN	Xp11.2 RCC	pT1bN0M0, I	IT	38, Normal
27	45/M/R	Symptomless	5.5	LNSS	Xp11.2 RCC	pT1bNxM0, I	IT	14, Normal
28	25/F/R	Gross hematuria	3.5	LRN	Xp11.2 RCC	pT1aNxM0, I	IT	13, Normal
29	39/F/R	Flank pain	4.5	LNSS	Xp11.2 RCC	pT1bNxM0, I	IT	11, Normal
30	45/F/L	Symptomless	12	ORN	Xp11.2 RCC	pT3aN0M0, III	TMT	30, Recurred in 12 months
31	30/F/L	Symptomless	9.5	LRN	Xp11.2 RCC	pT3aN0M0, III	TMT	20, Recurred in 14 months
32	64/M/L	Symptomless	3	LNSS	Xp11.2 RCC	pT1aN0M0, I	IT	3, Normal
33	55/F/R	Symptomless	3	LNSS	Xp11.2 RCC	pT1aN0M0, I	IT	8, Normal
34	42/M/L	Symptomless	3.5	LNSS	Xp11.2 RCC	pT1aN0M0, I	IT	4, Normal

ACJJ: American Joint Committee on Cancer; F: Female; IT: Immune therapy; L: Left; LNSS: laparoscopic nephron-sparing surgery; LRN: Laparoscopic radical nephrectomy; M: Male; ORN: Open radical nephrectomy; R: Right; RA: Radiofrequency Ablation; TMT: Targeted molecular therapy; VCTER: Vena cava tumor embolus resection.

### Evaluation of clinical data

The clinical data consisted of epidemiological features (gender and age), clinical manifestations, general history, preoperative CT imaging (laterality, tumor size and boundary), treatment methods (surgery, immune therapy or targeted therapy), pathological features (TNM stages, TFE3-IHC staining and FISH results) and clinical outcomes (normal, recurrent or dead). All of these data for the 34 patients were retrospectively analyzed. TNM staging was reclassified in accordance with the 7^th^ American Joint Committee on Cancer (AJCC) staging criteria (2010). All of the patients were followed every 3 months during the first year, every 6 months during the following 4 years, and annually after 5 years until the time of death or loss to follow-up.

### Statistical analyses

Progression-free survival (PFS) was defined from the initiation of surgery to the date of disease progression or censoring at the time of last follow-up. Overall survival (OS) was defined as the time interval between the date of surgery and the date of death or last follow-up. PFS and OS curves were obtained by Kaplan–Meier analysis, and statistical comparisons were performed using the log-rank test. Multivariate Cox regression model was used to evaluate the predictive role of the factors that showed significance in the long-rank test. Univariate and multivariate analyses were performed using SPSS software version 16.0 (SPSS Inc., Chicago, IL, USA). GraphPad Prism software version 5.0 was used to generate the survival curves. P-values <0.05 were considered statistically significant.

## Results

In total, 34 cases were identified encompassing 13 males and 21 females with a median age of 27 years (range of 3–64 years). Females with Xp11.2 tRCC in the age range of 18–45 years accounted for 81% of the patient population (17/21). With the exception of one patient who had a history of microcarcinoma thyroid in the previous 2 years, none of the patients had a history of tumors or chemotherapy. Nine of 34 (26%) patients underwent laparoscopic nephron-sparing surgery (LNSS) for the primary tumor, and the remaining 25 (74%) patients underwent open radical nephrectomy (ORN) or laparoscopic radical nephrectomy (LRN). Three of these latter patients who had inferior vena cava tumor thrombosis (cases 5, 6 and 10) underwent vena caval tumor thrombectomy simultaneously. The preoperative CT images for patients who underwent LNSS showed an isolated mass with a small diameter (<7 cm) and well-defined boundary.

Postoperative AJCC staging showed that 25 cases were classified as stage I/II, while 9 cases were classified as stage III/IV. Thirty-four patients were followed with a mean time of 41 months (range was 3–104 months). Twenty-four of the stage I/II cases received satisfactory results during a mean follow-up time of 42 months, except for one stage I male who was diagnosed with lung metastasis at the 11^th^ postoperative month. However, for the stage III/IV patients, 7 adults who received surgery and postoperative adjuvant molecular-targeted therapy (receptor tyrosine kinase inhibitor sunitinib or sorafinib) developed distant metastases or local recurrence, while two children who underwent surgery alone did not show disease progression during 65 and 71 months of follow-up. Of the three recurrent patients, case 25 was found abdominal wall recurrence, and case 30 along with case 31 were found ipsilateral abdominal cavity recurrence. Secondary surgeries were preformed to resect the recurrent lesions. Unfortunately, there was still one patient (case 25) who experienced an additional recurrence. Importantly, 9 patients who underwent LNSS showed no evidence of progress with a mean follow-up of 20 months.

Of the 34 cases diagnosed as Xp11.2 tRCC by the use of FISH, 7 cases (21%) were originally misdiagnosed as CCRCC (2 cases), PRCC (4 cases), and unclassified renal cell carcinoma (1 case).

**Fig 1:**
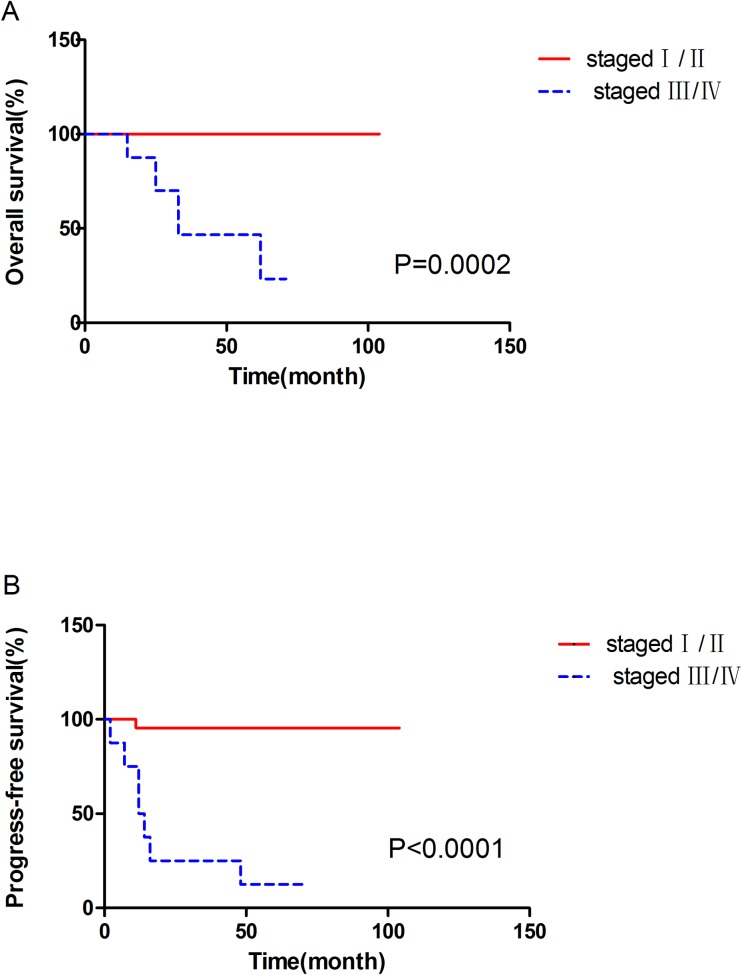
**Survival curves of overall survival (A) and progression-free survival (B) between stage I/II and stage III/IV patients.** The overall survival and progression-free survival between stage I/II and stage III/IV in Xp11.2 tRCC were statistically significant.

**Fig 2:**
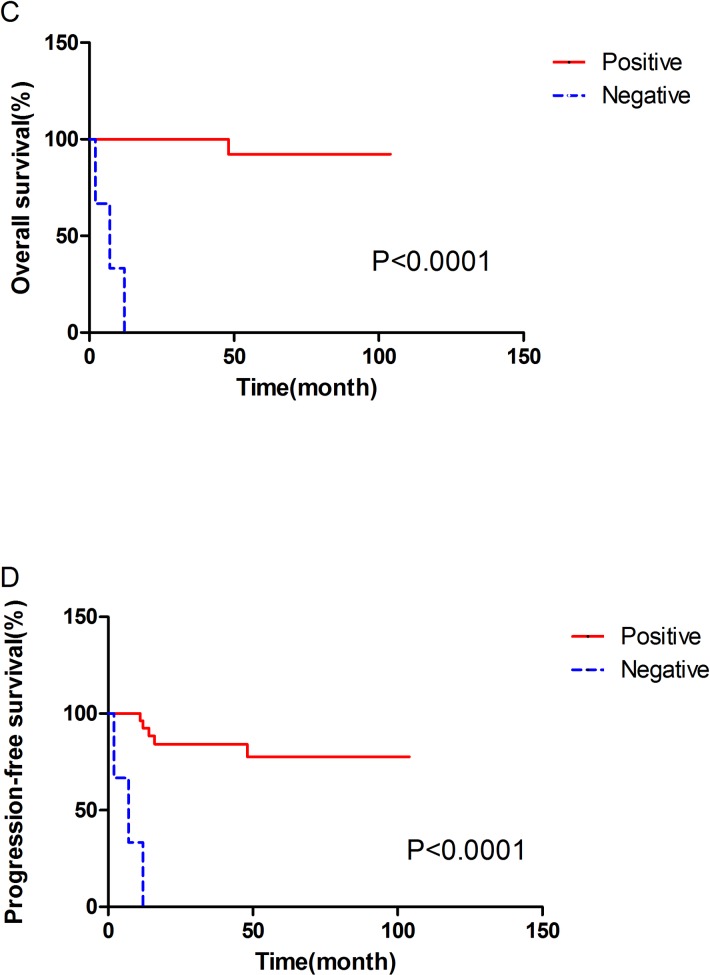
**Survival curves of overall survival (C) and progression-free survival (D) between inferior vena cava tumor thrombosis-positive and -negative patients.** The overall survival and progression-free survival between inferior vena cava tumor thrombosis-positive and -negative patients were statistically significant.

Table 2 summarizes each tested variable and the significance of variation. Univariate analysis showed that adjuvant treatment, TNM stage, regional lymph node metastasis and vena cava tumor thrombosis were risk factors for OS. In addition, maximum diameter, adjuvant treatment, TNM stage and vena cava tumor thrombosis were identified as risk factors for PFS (all P<0.05). The Kaplan-Meier survival curves for OS and PFS according to TNM stage are shown in Fig 1. The OS and PFS curves according to vena cava tumor thrombosis are shown in Fig 2. The four pairs of PFS and OS curves depending on TNM stage and vena cava tumor thrombosis were distinctly tiered and statistically significant.

**Table 2:** Kaplan-Meier univariate analysis of prognostic factors for overall survival (OS) and progression-free survival (PFS).

Variable		Number (%)	One-year overall survival rate	Three-year overall survival rate	Five-year overall survival rate	P value (Long-rank test)	
						OS	PFS
Age						0.230	0.192
	<18 years	4(11.8%)	100%	100%	100%		
	≥18 years	30(88.2%)	96%	84%	60%		
Sex						0.182	0.554
	Male	13(38.2%)	100%	100%	100%		
	Female	21(61.8%)	94%	86%	81%		
Symptom						0.753	0.211
	Gross hematuria	13(38.2%)	100%	90%	68%		
	Non-gross hematuria	21(61.8%)	93%	84%	84%		
Laterality						0.172	0.967
	Left	13(38.2%)	94%	81%	61%		
	Right	21(61.8%)	100%	100%	100%		
Operation						0.488	0.179
	Radical nephrectomy	25(73.5%)	96%	86%	69%		
	Partial nephrectomy	9(26.5%)	100%	100%	100%		
Maximum diameter						0.199	**0.007**
	≤7 cm	26(76.5%)	95%	95%	74%		
	>7 cm	8(23.5%)	100%	60%	60%		
Adjuvant treatment						**0.016**	**<0.001**
	Without	9(26.5%)	100%	100%	/		
	Immune therapy	15(44.1%)	100%	100%	/		
	Targeted molecular therapy	10(29.4%)	89%	64%	32%		
TNM stage						**0.002**	**<0.001**
	Staged I / II	25(73.5%)	100%	100%	100%		
	Staged III / IV	9(26.5%)	88%	59%	29%		
Lymph node metastasis						**0.018**	0.080
	Positive	5(14.7%)	100%	60%	30%		
	Negative	29(85.3%)	95%	95%	95%		
Inferior vena cava tumorthrombosis						**<0.001**	**<0.001**
	Positive	3(8.8%)	67%	/	/		
	Negative	31(91.2%)	100%	100%	82%		
Fusion type						0.280	0.439
	ASPL	6(26.1%)	100%	78%	39%		
	Non-ASPL	17(73.9%)	94%	87%	87%		
Boundary						0.088	**0.048**
	Clear	15(44.1%)	100%	100%	100%		
	Not clear	19(55.9%)	94%	80%	48%		

Multivariate analysis was performed using a Cox proportional hazards model to assess the independent prognostic value of clinicopathological features for PFS and OS. Considering that adjuvant treatment was closely associated with postoperative TNM stage, we excluded it when performing multivariate analysis to obtain a more reliable result. The Cox regression analysis result for PFS is shown in Table 3. TNM stage (P = 0.015) and vena cava tumor thrombosis (P = 0.024) showed independent prognostic significance. However, Cox regression indicated no significant independent prognostic factor when analyzing OS.

**Table 3:** Multivariate Cox regression analyses of progression-free survival.

Variable	B	SE	Wald value	P value	HR (95%CI)
Maximum diameter	-0.056	0.159	0.124	0.725	0.693–1.291
TNM stage	3.102	1.270	5.962	**0.015**	1.844–268.066
Lymph node metastasis	-1.492	1.003	2.212	0.137	0.031–1.607
Inferior vena cava tumor thrombosis	2.792	1.233	5.129	**0.024**	1.456–182.861
Boundary	11.613	234.618	0.002	0.961	0.000–5.635

## Discussion

Xp11.2 tRCC typically affects children and young adults under 45 years of age [3, 4, 19, 20] with a one-third incidence in juveniles and 0.2–5.0% incidence in adults [16, 21–23]. Unlike pediatric RCCs, cytogenetics is not routinely performed for adult RCCs due to the relatively lower incidence rate, which results in most misdiagnoses as conventional RCCs. Therefore, the exact frequency in adults is underestimated [21, 22], as inferred from our investigation. Compared with standard cytogenetics, TFE3-IHC has advantages of rapid diagnosis, high economy and high sensitivity in diagnosing Xp11.2 RCC. Thus, TFE3-IHC can be performed proactively as a screening test, and FISH can be performed as a verification test. Hirobe and Masumori suggested that the combination of TFE3-IHC and FISH is an effective method to diagnose Xp11.2 tRCCs, which can improve specificity and potentially eliminate false-positives resulting from overstaining [4].

In the current study, a predominance of young females and right side prevalence was observed, which is consistent with previous reports [4, 22, 24]. In the report by Argani on 28 cases of Xp11.2 tRCC, there was a strong female (22 cases, 79%) and a slight right (14/22, 64%) preference, of which 22 cases (79%) were younger than 45 years [22]. Choueiri et al. identified 15 Xp11.2 tRCCs, and female patients accounted for 80% [24]. Qu diagnosed 30 cases of Xp11.2 tRCC and reported that 18 cases (60%) were females, with 1/3 aged between 18 and 45 years and 17 of 30 cases showing a right side prevalence [2]. One probable explanation for the female predominance might be that females possess two X chromosomes while males only possess one, which leads to a higher incidence of X chromosome translocation. It remains unknown why the right side showed predominance. Due to an insufficient sample size, the actual frequency and mechanisms require further investigation.

With its aggressive biological behavior and local invasion tendency in adult, Xp11.2 tRCC trends to present with lymphatic and distant organ metastasis at diagnosis. To date, there is not yet a consensus regarding therapy, RN is recommended if possible [8]. Nevertheless, with the popularly utilized of NSS in recent year, part of the early stage Xp11.2 tRCC patients received NSS. Urologists and patients usually puzzled with the management between a completion nephrectomy and close surveillance when a patient was incidentally diagnosed with Xp11.2 tRCC after a partial nephrectomy. However, results from published studies reveal that the practice of NSS also produces favorable treatment outcomes in short-term follow-up studies [3, 18, 25]. Gorin and Ball reported that 4 patients who were treated with NSS remained alive without evidence of disease during a mean follow-up of 37 months [18]. Lim and You retrospectively analyzed 8 cases of Xp11.2 tRCC, and all of these patients underwent open or robotic NSS and were disease-free with a follow-up time of 18 to 47.6 months [3]. Herein, the treatment of Xp11.2 RCC by LNSS obtained a satisfactory result with a mean follow-up of 20 months. Even though, long-time follow-up is need as its well documented ability of recurrent late, especially PRCC-TFE3 RCC [5, 26].

Because pathological types are usually uncertain before surgery, radiologic and ultrasonic information may be the only reference to determine whether RN or NSS should be performed if a patient has a renal tumor. According to the literature, isolated masses with small diameters (<7 cm) and well-defined boundaries are regarded as appropriate candidates for NSS [17, 20, 27, 28], which is based on the finding that tumors with small diameters and clear rims predicate positive prognosis. From our statistical outcomes, both small diameters (<7 cm) and clear boundaries were protective factors for prognosis. Furthermore, Xiangming and colleagues reported that there was an inverse correlation between diameters and boundary, with larger diameters showing more indefinite boundaries [25]. As NSS offers oncological outcomes and preserves renal function comparable with that of RN, NSS should be considered if a young patient presents with a small lesion (<7 cm) and distinct boundary, especially patients with anatomical or a functional solitary kidney.

Since chemotherapy and radiotherapy proved to be invalid for Xp11 tRCC, the optimal and reliable adjuvant treatment remains to be developed. According to several previous studies, interferon (IFN-a) and interleukin-2 (IL-2) do not produce a significant response in this type of tumor [21, 29]. Recently, the molecular-targeted therapy using a receptor tyrosine kinase inhibitor, e.g., sunitinib or sorafinib, was shown to be a treatment choice for patients with lymph node or distant metastases [24, 30]. Choueiri et al. retrospectively reviewed 15 adult patients with metastatic Xp11.2 tRCC, and they reported that 3 patients achieved a partial response, 7 patients had stable disease, and 5 patients developed progressive disease [24]. However, the patients in Choueiri’s study were diagnosed by TFE3-IHC. In the study of Malouf, the researchers examined 21 cases of metastatic Xp11.2 tRCCs who received targeted therapy, and they reported that 7 patients achieved an objective response to sunitinib with a median PFS of 8.2 months compared to cytokine therapy with a median PFS of 2 months. In the current study, a 35-year-old male presented with lung metastasis at 11 months after surgery, and this patient then received targeted therapy for 8 weeks and survived during the postoperative follow-up of 50 months. However, all stage III adults who received ORN/LRN and targeted molecular therapy ultimately developed terminal or recurrent cancer. Giving the toxicity to growth, the usage of targeted therapy on juvenilities is usually limited, although trials have been reported [30, 31].

The literature shows that adults with Xp11 tRCC possess a grimmer prognosis than children, which is congruent with the data from our study, despite our finding from the univariate analysis indicated that age was not associated with prognosis due to the small sample size. For pediatrics, regional lymph node metastases do not necessarily predict poor prognosis [20, 23, 28, 32]. Geller et al. retrospectively reviewed 58 patients and found that children with lymph node-positive RCC in the absence of distant metastatic disease had a relatively favorable long-term prognosis with a survival rate (72.4%) nearly triple that of the adult controls [33]. Song et al. studied 22 cases of pediatric Xp11.2 tRCCs, including 12 stage III/IV patients, and reported that 13 patients were still alive and 2 had a terminal course with a postoperative follow-up of 6 months to 35 years [20]. Nevertheless, a meta-analysis showed that Xp11.2 tRCCs showed a poorer prognostic than non-Xp11.2 translocation carcinomas in children and young adults [34].

Similar to conventional RCCs, advanced TNM stage and inferior vena cava tumor thrombosis are the most significant factors that predict poor prognosis in Xp11 tRCC, which was verified by univariate and multivariate analyses in our study. Recently, Qu et al. [4] reported 30 cases of Xp11.2 tRCC diagnosed by FISH and showed that 11 of 14 stage III/IV patients presented with metastases of lung, liver or other organs, which was in contrast to the remaining 16 stage I/II patients. In the reports of Nesbitt et al., 20 of 37 patients with RCC with inferior vena cava tumor thrombus were alive after surgical resection, and the overall 2- and 5-year survival rates were 61.7% and 33.6%, respectively [35]. Nevertheless, the three patients with inferior vena cava tumor thrombosis in our investigation showed distant metastases and rapid termination of their life with a mean follow-up of 33 months, regardless of having undergone vena caval tumor thrombectomy and targeted molecular therapy.

In our investigation, 2 out of 6 ASPL-TFE3 RCC patients died due to multiple metastases. In the study by Ellis et al. [5], all of the published ASPL-TFE3 and PRCC-TFE3 cases were reviewed, and they reported that 24 out of 32 ASPL-TFE3 cases showed regional lymph node involvement, which was a higher incidence rate than that of PRCC-TFE3 cases. In the study by Komai et al. [36], 2 cases with ASPL-TFE3 RCC displayed metastasis, and one patient died of the disease. However, comparison of the survival time of Xp11.2 tRCC between ASPL fusion and non-ASPL fusion cases showed no difference by long-rank test in our study.

As its low-frequency, the reported Xp11.2 tRCCs which were diagnosed by FISH or PCR less than 150 all over the world. To date, this is one of the largest single-center clinical report on Xp11.2 tRCC confirmed by FISH. The data we published will undoubtedly enrich the literature base. There are some limitations to the current study. First, the sample size was insufficient due to the low incidence of this rare disease. Although the TFE3-IHC assay is sensitive to detect TFE3 in the screening of Xp11.2 tRCC, potential missed diagnoses may still exist. Second, the follow-up time was relatively short, especially for patients treated with NSS. Long-term follow-up is needed to assess the therapeutic effects of NSS. Third, only 6 cases were identified as genetic fusion types by the ASPL-TFE3 dual-fusion FISH assay, which did not allow the comparison between different subtypes of Xp11.2 tRCC to be performed. We are currently utilizing additional types of fusion probes to identify the genetic fusion types of those cases.

## Conclusions

Xp11.2 tRCC is a rare subset of RCC that mainly occurs in children and young females with gross hematuria. The combination of the TFE3-IHC assay and FISH analysis is an accurate and effective approach to screen and confirm, respectively, the diagnosis of Xp11.2 tRCC. Radical and partial nephrectomy constitute alternative treatments for patients with well-defined lesions measuring <7 cm. The prognosis of adults is significantly inferior to that of children, especially for patients at advanced TNM stages and with inferior vena cava tumor thrombosis.

## Supporting Information

S1 Table The STROBE checklist of the current study. This checklist contains items that reflected in our study according to STROBE guidelines.
(DOCX)
