# Supplementary material for: Renal Cell Carcinoma Associated with Xp11.2 Translocation/TFE3 Gene Fusions: Clinical Features, Treatments and Prognosis
Source: PLoS One. 2016 Nov 28;11(11):e0166897. doi: 10.1371/journal.pone.0166897 (PMC5125634; doi:10.1371/journal.pone.0166897)
Supplement: S1 Table — This checklist contains items that reflected in our study according to STROBE guidelines. (DOCX) [file pone.0166897.s001.docx]

**S1 Table. The STROBE checklist of the current observational study.**

|  | Item No. | Recommendation | Page  No. | Relevant text from manuscript |
| --- | --- | --- | --- | --- |
| **Title and abstract** | 1 | (*a*) Indicate the study’s design with a commonly used term in the title or the abstract | 1-2 | To investigate the clinical characteristics, treatments and prognosis of renal cell carcinoma associated with Xp11.2 translocation/TFE3 gene fusions (Xp11.2 tRCC), the clinical features and treatment results of 34 cases of Xp11.2 tRCC were retrospectively reviewed. |
|  |  | (*b*) Provide in the abstract an informative and balanced summary of what was done and what was found | 2 | Xp11.2 tRCC is a rare subtype of renal cell carcinoma that mainly occurs to young females. Radical nephrectomy and nephron-sparing surgery produced parallel outcomes in the treatment of small Xp11.2 tRCCs with clear rim in short-term follow-up. Advanced TNM stage and inferior vena cava tumor thrombosis indicated poor prognosis. |
| Introduction | | | |  |
| Background/rationale | 2 | Explain the scientific background and rationale for the investigation being reported | 3-4 | The diagnosis of Xp11.2 tRCC is difficult by histological characters and TFE3-IHC. TFE3 break-apart FISH is an ideal way. The implement of nephron-sparing surgery in Xp11.2 RCC was rare reported. |
| Objectives | 3 | State specific objectives, including any prespecified hypotheses | 4 | To investigate the effectiveness of nephron-sparing surgery in Xp11.2 RCC. On the other hand, survival rate and survival curve were analysed to finding out prognostic factors . |
| Methods | | | |  |
| Study design | 4 | Present key elements of study design early in the paper | 4-5 | The accurate diagnosis of Xp11.2 RCC |
| Setting | 5 | Describe the setting, locations, and relevant dates, including periods of recruitment, exposure, follow-up, and data collection | 6 | RCC patients were reviewed at Nanjing Drum Tower Hospital from January 2007 to February 2016.The review included their preoperative computed tomography (CT) characteristics, medical records, follow-ups and outcomes IHC stained was performed on formalin-fixed paraffin-embedded tissue sections. Then polyclonal break-apart probes for assay TFE3 gene rearrangement at the Xp11.2 region was performed on the patients who had a positive TFE3-IHC result on formalin-fixed paraffin-embedded tissue microarray slides. |
| Participants | 6 | (*a*) *Cohort study*—Give the eligibility criteria, and the sources and methods of selection of participants. Describe methods of follow-up.  *Case-control study*—Give the eligibility criteria, and the sources and methods of case ascertainment and control selection. Give the rationale for the choice of cases and controls  *Cross-sectional study*—Give the eligibility criteria, and the sources and methods of selection of participants | 5-6 | The patients who showed a positive result of fluorescence in situ hybridization were enrolled in the study. All the Xp11.2 RCC patients were followed every three months during the first year, every 6 months during the following four years, and annually after five years till the time of death or loss of follow-up. |
|  |  | (*b*) *Cohort study*—For matched studies, give matching criteria and number of exposed and unexposed  *Case-control study*—For matched studies, give matching criteria and the number of controls per case | 8-9 | The number of exposed and unexposed was displayed on **Table 2**. |
| Variables | 7 | Clearly define all outcomes, exposures, predictors, potential confounders, and effect modifiers. Give diagnostic criteria, if applicable | 6-7 | The TNM staging was reclassified in accordance with the 7th American Joint Committee on Cancer staging criteria. Progress- free survival was defined from the initiation of surgery to the date of disease progression or censoring at the time of last follow-up. Overall survival (OS) was defined as the time interval between the date of surgery and the date of death or last follow-up. |
| Data sources/ measurement | 8* | For each variable of interest, give sources of data and details of methods of assessment (measurement). Describe comparability of assessment methods if there is more than one group. |  |  |
| Bias | 9 | Describe any efforts to address potential sources of bias | 9-10 | All the Xp11.2 RCCs were identified by both IHC and FISH. When performing multivariate analysis adjuvant treatment was excluded considering it was closely associated with postoperative TNM stage, even it showed statistically significant in univariate analysis. One girl was lost to follow-up at 88 months. |
| Study size | 10 | Explain how the study size was arrived at | N/A | This kinds of renal cell carcinoma was rare, sample size was usually deficient. |

Continued on next page

| Quantitative variables | 11 | Explain how quantitative variables were handled in the analyses. If applicable, describe which groupings were chosen and why | 8-9 | Kaplan–Meier analysis and statistic comparison were undertaken using the log-rank test. In this case, quantitative variables was separated into groups. Which were shown on **Table 2**. |
| --- | --- | --- | --- | --- |
| Statistical methods | 12 | (*a*) Describe all statistical methods, including those used to control for confounding | 6-7 | Kaplan–Meier analysis and statistic comparison were undertaken using the log-rank test. Multivariate Cox regression model was used to evaluate the predictive role of the factors that show significant on long-rank test. |
|  |  | (*b*) Describe any methods used to examine subgroups and interactions | 7 | Multivariate Cox regression model |
|  |  | (*c*) Explain how missing data were addressed | 5-6 | Except a 11 years old girl was loss to follow-up, the clinical data were integrated, which was summarized in **Table 1.** |
|  |  | (*d*) *Cohort study*—If applicable, explain how loss to follow-up was addressed  *Case-control study*—If applicable, explain how matching of cases and controls was addressed  *Cross-sectional study*—If applicable, describe analytical methods taking account of sampling strategy | N/A | In our investigation, a 11 years old girl was lost to followed in 88 months postoperation. When analysing the epidemiology, the missing data was enrolled. When analysing prognostic factors, this case was regarded a patients which was followed 88 months with a normal resut. |
|  |  | (*e*) Describe any sensitivity analyses | N/A | N/A |
| Results | | | | |
| Participants | 13* | (a) Report numbers of individuals at each stage of study—eg numbers potentially eligible, examined for eligibility, confirmed eligible, included in the study, completing follow-up, and analysed | 4-5 | Of the 1239 RCC patients, 82 cases showed a positive reaction to TFE3-IHC, and 34 cases were eventually diagnosed as Xp11.2 tRCC by TFE3-IHC |
|  |  | (b) Give reasons for non-participation at each stage | N/A | Non-participation showed negative responses to TFE3-IHC andFISH. |
|  |  | (c) Consider use of a flow diagram | Unnecessary | Unnecessary |
| Descriptive data | 14* | (a) Give characteristics of study participants (eg demographic, clinical, social) and information on exposures and potential confounders | 7 | In total, 34 cases were identified in 13 females and 21 males with a median age of 27 years (range was 3-64 years). Females of Xp11.2 tRCC in the age range of 18-45 years accounted for 81% (17/21). |
|  |  | (b) Indicate number of participants with missing data for each variable of interest | 5-6 | The clinical data that used in our study was sufficient, which was displayed on **Table 1**. |
|  |  | (c) *Cohort study*—Summarise follow-up time (eg, average and total amount) | 7 | 34 patients were followed with a mean time of 41 months (range was 3- 104 months). |
| Outcome data | 15* | *Cohort study*—Report numbers of outcome events or summary measures over time | 9-10 | Report numbers of outcome events can be reflected by survival rate and survival curve. |
|  |  | *Case-control study—*Report numbers in each exposure category, or summary measures of exposure |  |  |
|  |  | *Cross-sectional study—*Report numbers of outcome events or summary measures |  |  |
| Main results | 16 | (*a*) Give unadjusted estimates and, if applicable, confounder-adjusted estimates and their precision (eg, 95% confidence interval). Make clear which confounders were adjusted for and why they were included | 10 | Multivariate Cox regression was shown on **Table 3**, P value and 95% CI was clear from the table. |
|  |  | (*b*) Report category boundaries when continuous variables were categorized | 9-10 | Only age and maximum diameter were continuous variables in our study. Age boundaries was 18 years old between children and adults. According to the tumours sizes on preoperation, patients was separated into maximum diameter over 7 cm and not more than 7cm. |
|  |  | (*c*) If relevant, consider translating estimates of relative risk into absolute risk for a meaningful time period | N/A | N/A |

Continued on next page

| Other analyses | 17 | Report other analyses done—eg analyses of subgroups and interactions, and sensitivity analyses | 8-9 | Kaplan–Meier analysis and statistic comparison were undertaken using the log-rank test. In this case, quantitative variables was separated into groups. Which were shown on **Table 2**. |
| --- | --- | --- | --- | --- |
| Discussion | | | | |
| Key results | 18 | Summarise key results with reference to study objectives | 10-15 | A predominance of young females and right sides were displayed. Our preliminary attempt to treat Xp11.2 RCC by using LNSS got a satisfied result with a mean follow-up of 20 months. Advanced TNM stage and inferior vena cava tumor thrombosis are the most significant factors that predict poor prognosis in Xp11 tRCC |
| Limitations | 19 | Discuss limitations of the study, taking into account sources of potential bias or imprecision. Discuss both direction and magnitude of any potential bias | 15 | The sample size is still insufficient due to the low incidence of this rare disease. The follow-up time is relatively short especially the patient treated by NSS. Only 6 cases are identified genetic fusion types ASPL-TFE3 dual-fusion FISH assay, which makes the comparison between different subtypes of Xp11.2 tRCC cannot be performed. |
| Interpretation | 20 | Give a cautious overall interpretation of results considering objectives, limitations, multiplicity of analyses, results from similar studies, and other relevant evidence | 15 | Conclusions |
| Generalisability | 21 | Discuss the generalisability (external validity) of the study results | N/A | Xp11.2 tRCC was a special type of renal cell carcinoma. |
| Other information | |  | | |
| Funding | 22 | Give the source of funding and the role of the funders for the present study and, if applicable, for the original study on which the present article is based | 15 | Acknowledgements |

*Give information separately for cases and controls in case-control studies and, if applicable, for exposed and unexposed groups in cohort and cross-sectional studies.

**Note:** An Explanation and Elaboration article discusses each checklist item and gives methodological background and published examples of transparent reporting. The STROBE checklist is best used in conjunction with this article (freely available on the Web sites of PLoS Medicine at http://www.plosmedicine.org/, Annals of Internal Medicine at http://www.annals.org/, and Epidemiology at http://www.epidem.com/). Information on the STROBE Initiative is available at www.strobe-statement.org.
